# Survival from cardiopulmonary arrest after regular working hours in a tertiary-care hospital: retrospective study

**DOI:** 10.1186/cc12242

**Published:** 2013-03-19

**Authors:** A Mir, A Hussein, A AlEnezi, M Almaani, M Shah, A Shah, B Daoud

**Affiliations:** 1 King Fahd Medical City, Riyadh, Saudi Arabia

## Introduction

Detection and treatment of cardiopulmonary arrest and their antecedents may be less effective at night and weekend than weekdays because of hospital staffing and response factors [1]. Early detection and resuscitation of cardiopulmonary arrest are crucial for better clinical outcome. We conducted our study to evaluate event survival of in-hospital cardiopulmonary arrest after regular working hours in nonmonitored areas of a tertiary-care center.

## Methods

A retrospective chart review of all adult patients who developed in-hospital cardiopulmonary arrest between January 2010 and December 2011. Working hours are defined as 07:00 to 17:00 Saturday to Wednesday. Event survival is defined as return of spontaneous circulation (ROSC) for more than 20 minutes. Adult patients 18 years and above who suffered from in-hospital cardiopulmonary arrest were included. Patients were excluded if they had cardiopulmonary arrest in the emergency department, had implantable cardiovascular device, arrested in monitored areas or had pre-existing DNR orders. Data analysis was accomplished using SAS, version 9.3 (SAS institute, Inc., Cary, NC, USA).

## Results

A total of 430 cardiopulmonary arrest events occurred in hospital between January 2010 and December 2011. A total of 326 patients were excluded because 252 occurred in the ICU, 70 in the coronary care unit and four in the stork unit. In total, 104 patients were enrolled; 50.9% where female and 49.1% male. Median age was 61 years. A total 41.3% of the arrests were due to respiratory arrest and 58.7% due to cardiac arrest. Out of 104 patients who developed cardiopulmonary arrest, 47 (45.19%) occurred during regular working hours, and 57 (54.81%) occurred after regular working hours (*P *= 0.0081). The event survival was 87.23% during regular working hours compared with 47.37% for the patients who developed cardiopulmonary arrest after regular working hours (*P <*0.0001). In total, 53.19% of those who developed cardiopulmonary arrest during regular working hours were discharged alive from the ICU compared with 26.32% of those who developed cardiopulmonary arrest after regular working hours.

## Conclusion

Event survival and survival to discharge were significantly higher in patients who developed cardiopulmonary arrest during regular working hours.
